# The Oral Health Inequities between Special Needs Children and Normal Children in Asia: A Systematic Review and Meta-Analysis

**DOI:** 10.3390/healthcare9040410

**Published:** 2021-04-02

**Authors:** Valendriyani Ningrum, Abu Bakar, Tzong-Ming Shieh, Yin-Hwa Shih

**Affiliations:** 1 School of Dentistry, Baiturrahmah University, Padang 25586, Indonesia; valend888@gmail.com (V.N.); abuba.mmed@gmail.com (A.B.); 2 School of Dentistry, China Medical University, Taichung 404333, Taiwan; tmshieh@mail.cmu.edu.tw; 3 Department of Healthcare Administration, Asia University, Taichung 41354, Taiwan

**Keywords:** oral health inequity, special needs children, meta-analysis

## Abstract

This meta-analysis aimed to analyze the oral health inequalities among special needs children during 2004–2019 in Asia to reveal the importance and the needs of establishing integrated and equitable special needs dentistry care system in Indonesia. PubMed, Scopus, Cochrane Library, Web of Science, and Wiley Online Library were systematically searched for full-text observational studies published from 1 January 2004 to 15 January 2020, in English in Asia. Studies that included children under 18 years of age with special needs and compared them to healthy controls were selected. Study quality was assessed using the Joanna Briggs Institute 2017 Critical Appraisal Checklist. Risk of bias was assessed using the ROBINS-E tool. The decayed, missing, and filled permanent teeth (DMFT) index indicated that special needs children who suffer from intellectual disability or autism spectrum disorder had significantly more caries than normal children (*p* < 0.001). The special needs children who had more caries than normal children lived in countries that had a high average DMFT value among 12 years old children (*p* < 0.001), and these two variables showed a positive correlation in meta-regression analysis (*p* < 0.001). Having an integrated and equitable care system and elevating children’s oral health are important to maintain special needs children’s oral health.

## 1. Introduction

Special needs children are individuals under the age of 18 who have either physical or cognitive disabilities, including intellectual disability (ID), Down Syndrome (DS), autism spectrum disorder (ASD), cerebral palsy (CP), epilepsy (EP), and attention deficit hyperactivity disorder (ADHD) [1]. They have oral health problems similar to those of normal children, such as dental caries, poor oral hygiene, periodontal disease, and malocclusion [2,3,4,5,6,7,8,9,10,11,12,13,14,15,16,17,18,19,20,21,22,23,24,25,26,27,28,29,30].

Children with special needs have congenital developmental disorders that lead to them experiencing oral health inequality. Open bite and dysphagia usually occur in children with DS [31] and increased plaque and calculus formation give rise to poor oral hygiene [32]. In particular, individuals with ASD have poor dietary preferences, behaviors and specific aversions, bruxism, gingival picking, self-removal of teeth, chewing on hard items, and repeated regurgitation that may cause tooth avulsion [33] making them more susceptible to oral health problems [34].

Most children aged less than 7 years need caregivers to assist in maintaining oral hygiene in daily life. Children with special needs require more assistance even when they are over seven years of age due to their mental and physical challenges. Some special needs children learn slowly and often have difficulty understanding others’ behavior and their own, such as brushing teeth (mental challenges). Some of them have scoliosis, unsteady gait, or increased limb tone (physical challenges).

Children in Asia are more likely to have poor oral health if their caregivers have low-income, have a low educational level, live in rural areas, and have suboptimal access to quality oral health care. As a result, poor oral health among children reflects social inequity. Children with special needs show anxiety and uncooperative behavior during dental care and treatment (behavioral challenges), similar to normal children. Specific communication skills and sedative equipment to alleviate anxiety and uncooperative behavior are needed. As a consequence, there is reduced willingness among caregivers and non-trained dentists to provide oral health care to special needs children, resulting in oral health inequities [35].

Numerous articles have investigated the clinical oral health status of children with special needs to shed light on their unmet needs. The aim of this study was to provide evidence-based outcomes through systematic review and meta-analysis. This highlights the need for an integrated and equitable system for special-needs dentistry in Asia.

## 2. Materials and Methods

### 2.1. Data Sources and Searches

This systematic review was conducted and reported according to the Preferred Reporting Items for Systematic Reviews and Meta-Analyses (PRISMA) Meta-analysis of Observational Studies in Epidemiology (MOOSE) reporting guidelines [36,37,38]. We searched articles on PubMed (1369), Web of Science (310), Scopus (10), Wiley Online Library (633), and Cochrane Library (15 present) between 1 January 2004, and 15 January 2020 (Tables S1 and S2) (Supplementary Materials). Search keywords are given in the supplemental data (Figures S1–S5) (Supplementary Materials). The search included indexed terms and text words to capture the concept of clinical oral health assessment among children with special needs in Asia. Full-text research articles with cross-sectional designs published in English were included. For all the included studies, the participants provided written assent and parental informed consent.

### 2.2. Study Selection

We included cross-sectional studies that evaluated oral health status by quantitative measurement using the decayed, missing, and filled permanent teeth (DMFT) index, oral hygiene index-simplified (OHI-S), plaque index, community periodontal index, and treatment needs (CPITN) index, and gingiva index, in special needs children under 18 years of age and compared them with normal children. Two investigators (co-first authors) independently reviewed the title, abstract, and text of the articles. Studies were included when the kappa score assessed by both reviewers was above 0.91 for the selected study.

### 2.3. Critical Appraisal of Identified Studies

The appraisal reliability between the two reviewers was calculated using the Statistical Package for the Social Sciences software version 23.0, for Windows (IBM, New York, NY, USA). A kappa value above 0.91 indicated high reliability. Two reviewers (co-first authors) appraised inclusion studies with the Joanna Briggs Institute 2017 critical appraisal checklist for analytical cross-sectional studies (Table S3) (Supplementary Materials). The following eight criteria were used: (1) the criteria for inclusion in the sample were clearly defined; (2) the study subjects and the setting were described in detail; (3) the exposure was measured in a valid and reliable way; (4) objective, standard criteria were used to measure the condition; (5) confounding factors were identified; (6) strategies to deal with confounding factors were stated; (7) the outcomes were measured in a valid and reliable way; and (8) appropriate statistical analysis was used. The risk of bias of articles was appraised using the ROBINS-E tool (University of Bristol, Bristol, UK) (Table S4) (Supplementary Materials). 

### 2.4. Data Extraction

The country, disability type, age, sample size, key findings, comments, and mean and standard deviation of the DMFT index (13 present), plaque index (four present), OHI-S index (five present), CPITN index (two present), and gingival index (three present) were extracted from the articles (Tables S5–S9) (Supplementary Materials). The accuracy of data extraction was confirmed by the authors (co-first authors).

### 2.5. Statistical Analysis

Meta-analyses were conducted using Comprehensive Meta-Analysis Version 2 for Windows (Biostat, NJ, USA). Because both the diagnosis of special needs children and oral health status assessment have international standards, we believe that all the studies were functionally identical. Thus, we performed a meta-analysis assuming a fixed-effect model [39]. According to the Cochrane Handbook 5, the heterogeneity data could be ignored, and the test of the null hypothesis is meaningful under the fixed-effect model. Heterogeneity among the studies was assessed using *I^2^* statistics. We conducted meta-regression and subgroup analyses of the DMFT index [40]. The subgroup analysis was subsequently stratified by specific disability types, country, and the worldwide average value of DMFT in 12-year-olds (Figure S6) (Supplementary Materials). Statistically significant differences in the null hypothesis test was set at *p* < 0.05. The meta-regression was conducted by the moderator of the average value of DMFT in 12-year-olds (very low = 0.5, low = 1.8, moderate = 3, high = 3.5). Publication bias was assessed by visually inspecting the funnel plot for a skewed distribution and Egger’s test (Figure S7) (Supplementary Materials).

## 3. Results

### 3.1. Comprehensive Systematic Literature Search

**Figure 1:**
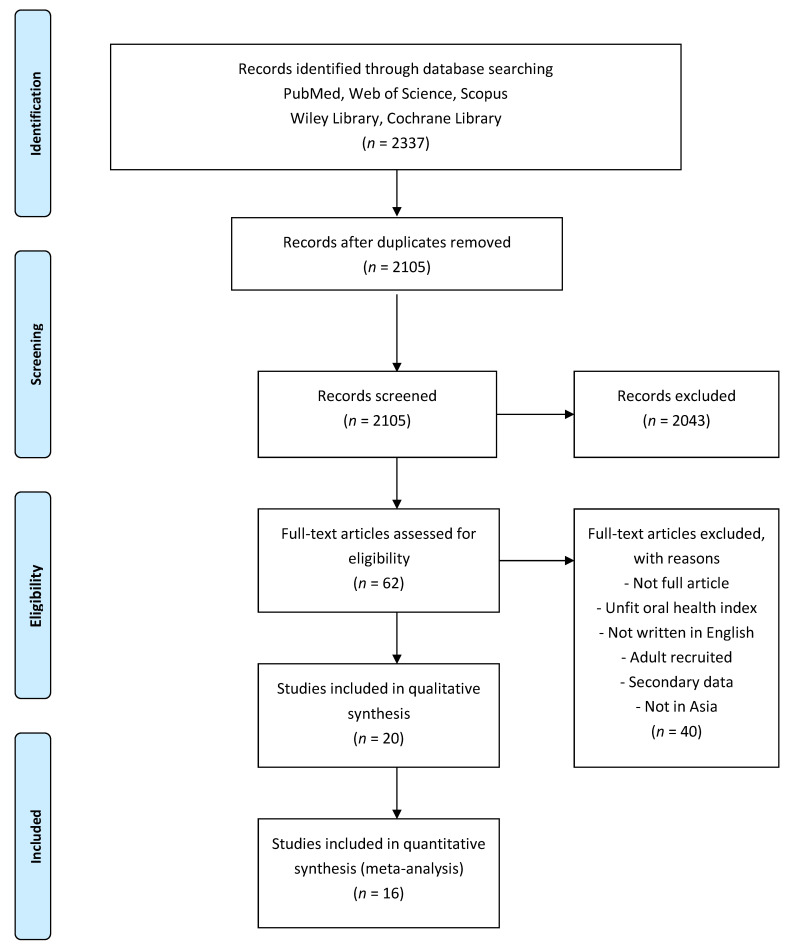
The PRISMA flow chart. There were 2337 articles identified from the web database and 2105 articles were left after duplicate removal. After the article appraisal procedure, 20 articles were left for systematic review, and 16 articles left for meta-analysis.

Of the 2105 studies identified, 20 met the inclusion criteria. Sixteen articles were included in the meta-analysis (Figure 1). We pooled the extracted data and analyzed the DMFT index (13 studies, 796 special needs children and 880 normal children), plaque index (four studies, 484 special needs children and 526 normal children), OHI-S index (five studies, 321 special needs children and 384 normal children), CPITN index (two studies, 241 special needs children and 253 normal children), and gingiva index (three studies, 224 special needs children and 376 normal children). The ages ranged from 2.6-18 years. Data on oral health status assessment were available from five countries: Korea (one study), China (two studies), Thailand (one study), India (eight studies), Pakistan (one study), United Arab Emirates (three articles), Yemen (one study), Turkey (one study), and Israel (two studies). The types of disabilities among children with special needs were DS, ASD, ADHD, ID, CP, and EP. The characteristics of the included studies are summarized in Table 1.

### 3.2. Pooled Oral Health Status Index Data

**Figure 2:**
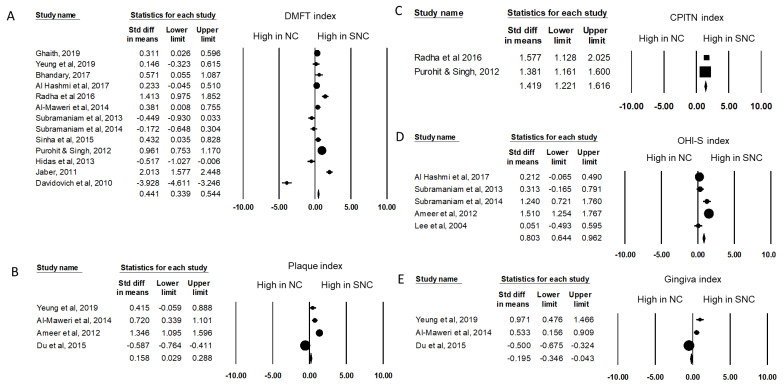
The pool extracted data of oral health index among children. (**A**) DMFT index (**B**) Plaque index (**C**) CPITN index (**D**) OHI-S index (**E**) Gingiva index. The forest plot revealed the standard difference in means of DMFT index (0.4441), Plaque index (0.158), CPITN index (1.419), and OHI-S index (0.803) were higher in special needs children. NC: normal children. SNC: special needs children.

We conducted a meta-analysis to compare the oral health status index between children with special needs and healthy children. The DMFT pooled data indicated that the standard difference in the means was 0.441 (95% CI: 0.339–0.544, *p* < 0.001) (Figure 2A). The pooled plaque index data indicated that the standard difference in means was 0.158 (95% CI: 0.028–0.288, *p* = 0.017) (Figure 2B). The CPITN index pooled data indicated that the standard difference in the mean was 1.419 (95% CI: 1.221–1.616, *p* < 0.001) (Figure 2C). The OHI-S index pooled data indicated that the standard difference in the means was 0.803 (95% CI: 0.644–0.962, *p* < 0.001) (Figure 2D). The gingiva index pooled data indicated that the standard difference in means was −0.195 (95% CI: −0.346–−0.043, *p* < 0.001) (Figure 2E). The publication bias and heterogeneity of the included studies are listed in Table 2. Of the five indices, the gingiva index showed publication bias. The outcomes of the meta-analysis revealed that the DMFT, plaque, CPITN, and OHI-S index values were significantly higher in children with special needs.

### 3.3. Subgroup Analysis and Meta-Regression

**Figure 3:**
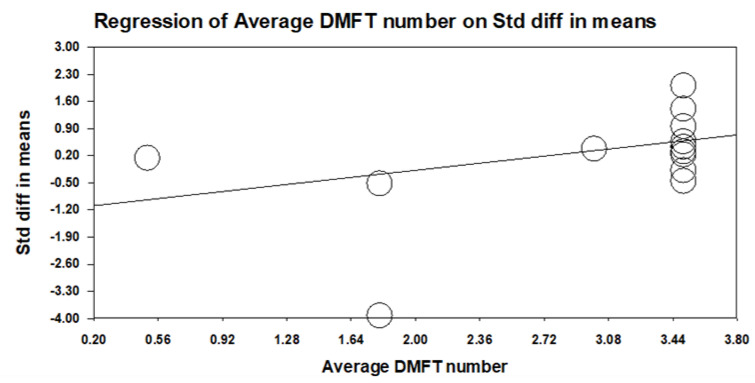
The meta-regression analysis of the average DMFT number in each country during 1994–2014. The standard difference in means of DMFT index in the forest plot was positively correlated with the 12-year-old children average DMFT number of the corresponding country during 1994–2014. B = 0.51, SE = 0.07, 95% CI: 0.37–0.65, *p* < 0.001.

Based on the observed high heterogeneity among pooled DMFT index studies, we con-ducted a series of subgroup analyses stratified by disability type, country, and average DMFT value reported in each country (Table 3). Children with ASD and ID had significantly higher numbers of caries than normal chil-dren (*p* < 0.001). Special needs children in India, the United Arab Emirates, and Yemen had a significantly higher number of caries than normal children (*p* < 0.05). This outcome was not observed in China (Hong Kong) or Israel. In countries with low average DMFT values, normal children had a significantly higher number of caries (*p* < 0.001), and in countries with moderate and high average DMFT values, special needs children had significantly higher caries numbers. The meta-regression showed a positive correlation between the DMFT standard difference in means and the average DMFT value in each country (B = 0.51, SE = 0.07, 95% CI: 0.37–0.65, *p* < 0.001) (Figure 3). The outcome indicated that the disability types and the variance in average DMFT value in each country affect the number of children with special needs. 

## 4. Discussion

This study identified oral health issues among children with special needs. Although the included 20 articles were selected after strict appraisal, four of them could not be pooled for meta-analysis because no mean and standard deviation of the oral health index values were shown (Table 1). Namal et al. [40] showed a DMFT index of more than 1 and 0, Luppanapornlarp et al. [42]. missed periodontal pocket depth data due to behavioral challenges of ASD participants, Rai et al. [20] showed OHI-S scores as the median value, and Suhaib et al. [15] showed oral health status based on the presence or absence of an oral health condition. The pooled data indicated that most oral health conditions among children with special needs were worse than those of normal children. Two included studies revealed significantly better oral health status among children with special needs. Davidovich et al. [41] showed that children with DS had lower DMFT values in Israel (Table S5) (Supplementary Materials), and Du et al. [48] showed that children with ASD had lower plaque index (Table S6) (Supplementary Materials) and gingiva index (Table S9) (Supplementary Materials) values in Hong Kong. We noticed that the average DMFT values among 12-year-old children in Israel and Hong Kong were low and very low, respectively (Table 1). We conducted an in-depth exploration of these articles. Davidovich et al. [41] thought that the salivary minerals of children with DS were the main factor protecting them from caries. However, this hypothesis could not be proven in the study by Ghaith et al. [53] in the United Arab Emirates, and the subgroup analysis found no significance in the pooled data (Table 3). Du et al. [48] did not conduct oral health examinations among a quarter of children with ASD with behavioral challenges. These children may have worse oral health, and nonresponse bias should be considered when interpreting the findings.

The main factors that contributed to the worse oral health status of children with special needs were mental challenges, behavioral challenges, physical challenges, congenital abnormalities of oral facial development, and side effects of medication. For children with mental challenges, health care providers should design special educational materials to teach children the concept of oral health and the importance of how to brush teeth adequately. For children with behavioral challenges, the main caregivers and dentists should learn appropriate skills for oral examination. Special dental clinics should have specific techniques to manage uncooperative behavior, such as restraining patient chairs and sedation or general anesthesia. For children with physical challenges, a proper assistive device designed for an individual’s needs would help them maintain oral hygiene. Open bite and Class III angle malocclusion were significantly more common in individuals with DS than in normal children. Children with CP have more frequent Class 2 angle malocclusion caused by an abnormal alignment of the tongue, lips, and cheeks along with oral habits. Children with EP who used more than one antiepileptic drug had a higher prevalence of dental caries than those who used mono-antiepileptic drug therapy. Combining congenital deficiencies, consumption of sweet diets, inadequate nutrition, and poor oral hygiene makes certain children highly susceptible to caries.

The main caregiver’s oral health literacy is reported to be associated with the oral health status of children with special needs. Parents’ oral health literacy is reported to be an important determinant of oral health-related expenditure [54]. The caregiver’s sex, educational level, perception of the children’s oral health, and family socioeconomic status were significant predictors of the children’s caries experience [55,56]. In addition, two-thirds of the caregivers had barriers to access dental care, such as waiting too long for a visit, treatment of the special needs children under dental surgery conditions (due to behavioral challenges), and lack of satisfaction with their dental care [57]. This will cause more untreated oral health problems among children with special needs [50]. Therefore, special needs dentistry should be widely popularized, provide adequate oral health knowledge to caregivers, and make the treatment more efficient, friendly, and more satisfactory by specially trained dentists.

The strengths of this study include a comprehensive search strategy, strict inclusion and exclusion criteria, critical appraisal, and analytical strategies that include subgroup analysis and meta-regression to identify heterogeneity among articles in depth. All included studies used the same diagnostic guidelines that minimized the confounding effect while pooling the data and analysis. This study has some limitations. Not all Asian countries have special needs dentistry. The prevalence of oral health problems among children with special needs may be underestimated. The overall risk of bias was moderate due to confounding factors, and the reported results were moderate. We pooled the extracted data from parts of Asian countries and different types of special needs children; therefore, our results may not apply to every group of children with special needs in every Asian country.

## 5. Conclusions

Our results indicated that oral health status is worse among special needs children compared to normal children, and this issue should be taken seriously to defend their right to health and well-being. Special needs children need more help with activities of daily living from their caregivers and proper oral health management by a specially trained dentist. In addition to promoting special needs dentistry clinics in every country, setting up a special dental clinic in special-care schools or arranging regular campus dental visits to assess proper dental treatment are also suggested. Policies should be made to improve the oral health status of children in high average DMFT countries, such as teaching children the proper way to brush teeth, removing dental plaque, avoiding sweet desserts to reduce acid production from germs, schedule regular campus dental visits for oral health education, early treatment of dental problems, and applying fluoride varnish to harden the enamel.

## Figures and Tables

**Table 1:** The characteristics of the included studies.

No.	Source	Country (DMFT dg)	Participants	Age	Sample Size	Covariate	Outcomes	Appraisal Comment
1	Lee et al., 2004 [39]	Korea (Low)	DS	8–17 years	19	dmfs, OHIs	OHIs Index and total salivary Ig A similar for both DS and general children, but significantly higher serotype g-s-IgA and serotype c-s-IgA in DS group.	This pioneering study suggests the relationship between the lower prevalence of caries in Down syndrome children and the higher *S. mutans*-specific IgA concentrations, although did not adequately explain the causal relationship
			N	8–17 years	41			
2 *	Namal et al., 2007 [40]	Turkey (Low)	ASD	7–12 years	62	DMFT	Children with ASD had better dental caries status than healthy children perhaps due to the ASD parents managing their children diet.	General information about dental caries due to these studies only showed DMFT percentage in two categories; DMFT > 1 and DMFT = 0.
			N	7–12 years	301			
3	Davidovich et al., 2010 [41]	Israel (Low)	DS	4.41 ± 1.9 years	70	PI, GI, DMFT	Sialo chemistry analysis showed calcium, sodium, potassium, and chloride levels were significantly higher in the DS population. The mean age of the study group is lower than the mean age of the control group.	Salivary ion expression is most influential in lower caries rates among DS children.
			N	9.22 ± 2.7 years	32			
4 *	Luppanapornlarp et al., 2010 [42]	Thailand	ASD	8–12 years	32	CPITN, DAI	ASD children had significantly poorer periodontal health than control group. Similar malocclusion was found between both groups. ASD children showed more diastema, spacing, missing teeth, open bites, reverse overjet, and Class II molar relationship than healthy individuals.	Due to ASD behavior challenges, pocket-depth is difficult to measure and these categories are missing in periodontal status.
		(Low)	N	8–12 years	48			
5	Jaber, 2011 [34]	United Arab Emirates (High)	ASD N	6–16 years	61	GI, OHIs, DMFT	ASD children showed higher caries rates, worse oral hygiene and dental treatment needs than healthy control group.	Oral hygiene status present in general percentage of ASD group and control group.
				6–16 years	61			
6	Hidas et al., 2013 [43]	Israel (Low)	ADHD	5–18 years	31	DMFT, PI	ADHD children showed similar caries rate, higher plaque index and hyposalivation compared with the control groups.	The study used self-reported questionnaires to assess oral hygiene behavior and the validity of the questionnaires is low.
			N	5–18 years	30			
7 *	Rai et al., 2012 [20]	India (High)	ASD	6–12 years	101	OHIs, dentition status index.	Similar dental caries status among ASD children and their siblings. In contrast, oral hygiene of ASD children worse than their siblings.	OHI-S score shows median value; median is generalized and difficult to manage theoretically.
			N	6–12 years	50			
8	Ameer et al., 2012 [44]	India (High)	ID	4–17 years	150	CPI, PI, OHIs	ID groups showed lower oral hygiene and higher periodontal disease, perhaps due to lack of understanding, coordination, or muscular limitations.	Using fingers as oral hygiene aid among several ID groups (48%) is uncommon.
			N	4–17 years	150			
9	Purohit & Singh, 2012 [4]	India (High)	Disability not specified	12 years	191	CPI, DAI, DMFT	Children with disabilities showed poorer oral hygiene with greater calculus deposition, 30% more caries rates and a 60% higher malocclusion compared to control group.	A study using participants, specifically 12-year-olds, withpermanent teeth, and appropriate to the WHO recommendations for international comparison and monitoring trends of toothache.
			N	12 years	203			
10	Subramaniam et al., 2014 [45]	India (High)	DS	7–12 years	34	DMFT, OHIs	DS children showed significantly lower total antioxidant capacity of saliva and higher salivary sialic acid levels. There was an inverse relationship between total anti-oxidant capacity and dental caries.	The study highlights the importance of saliva as a diagnostic tool for prevention of oral disease in high-risk individuals.
			N	7–12 years	34			
11	Sinha et al., 2015 [46]	India (High)	CP	7–17 years	50	DMFT, OHIs	Cerebral palsy subjects had higher caries and poor oral hygiene perhaps due to drooling problem from swallowing effect. CP children have greater Class 2 Angle’s malocclusion caused by abnormal alignment of the tongue, lips and cheeks along with oral habits.	The study cannot be generalized due to the small sample size and uncontrolled effect of cerebral palsy medication in oral health.
			N	7–17 years	50			
12	Subramaniam et al., 2013 [47]	India (High)	CP	7–12 years	34	DMFT, OHIs	Higher dental caries in CP children due to inconsistent diet, inadequate nutrition and poor oral hygiene.	This study emphasized the influence of oxidative stress and antioxidants on oral health particularly in cerebral palsy individuals.
			N	7–12 years	34			
13	Du et al., 2015 [48]	China -Hong Kong (Very low)	ASD	32–77 months	257	PI, GI, dmfs	ASD children had better gingival health and less caries prevalence than control subjects. Both groups showed similar prevalence of malocclusion.	The article title suggests this study is a case control study; however, it is a cross-sectional study.
			N	32–77 months	257			
14	Al-Maweri et al., 2014 [49]	Yemen (Moderate)	ASD	5–16 years	42	PI, GI, DMFT	ASD children have high prevalence of oral health problems such as poor oral hygiene, gingivitis, fistulae, ulcerative lesions, gingival hyperplasia and cheilitis. The DMFT score was not statistically significant; in contrast the DMFT score was significantly higher in ASD children than control group (5.23 vs. 4.06; *P* < 0.001).	Relatively small number of participants included this study due to limited number of ASD special schools in the area.
			N	5–16 years	84			
15	Radha et al., 2016 [50]	India (High)	ID	9–14 years	50	CPI, DMFT	ID children had higher value for Decay and Missing teeth, while general children had higher value for Filling teeth.	The study suggests that future studies conduct biochemical and microbiological analysis in a larger sample.
			N	9–14 years	50			
16	Al Hashmi et al., 2017 [51]	United Arab Emirates (High)	CP	4–18 years	84	DMFT, OHIs	Caries rate was similar between the CP and control subjects. CP subjects had significantly higher Class II malocclusion, anterior open bite, anterior spacing, and trauma in anterior teeth. In addition, higher frequencies of macroglossia and drooling.	The authors recommend systematic reviews to measure the oral health status of CP patients to provide important high‑quality evidence in this area.
			N	4–18 years	125			
17	Bhandary, 2017 [52]	India (High)	ASD	6–12 years	30	OHIs, DMFT	ASD children similar to their healthy siblings in caries score and OHIs index showed fair gingival bleeding.	Interestingly, the study discussed pro and contra literature regarding pH and buffering capacity differences between ASD children and healthy children.
			N	6–12 years	30			
18 *	Suhaib et al., 2019 [15]	Pakistan (Low)	ASD	2–10 years	58	Caries, periodontal disease clinical examination based on absence or presence of an oral condition	The mother’s education associated with dental caries and periodontal disease in ASD children. In addition, ASD demonstrated higher caries incidence and dental plaque on anterior teeth. Self-injurious behavior and bruxism showed in some ASD children.	The study was conducted with small sample size; hence these results cannot be generalized to the global population.
			N	2–10 years	27			
19	Ghaith, 2019 [53]	United Arab Emirates (High)	DS	4–18 years	84	Angle malocclusion classification, DMFT, OHIs	The DMFT Index, open bite and Class III Angle’s malocclusion were significantly higher in DS than healthy children.	Malocclusion and OHIs presented in general outcomes (percentages). Parent’s awareness is an important variable suggested for future special needs care dentistry study.
			N	4–18 years	112			
20	Yeung et al., 2019 [29]	China -Hong Kong (Very low)	EP	3–18 years	35	DMFT, PI, GI, Gingival overgrowth index.	Children with epilepsy showed significantly worse gingival health than control children. Epileptic children who consume more than 1 antiepileptic drug had a higher dental caries prevalence than those who use mono-antiepileptic drug therapy.	Comprehensive study presented oral health status related to drug use in epilepsy children
			N	3–18 years	35			

* = Excluded from meta-analysis. Ig A = Immunoglobulin A; g-s-IgA = g strain S. mutans-specific IgA; c-s-IgA = c strain S. mutans-specific IgA; dmfs = decayed, missing, and filled primary teeth or surfaces; OHIs = Oral Hygiene Index simplified; DMFT = Decay, Missing, Filled Permanent Teeth; PI = Plaque Index; GI = Gingival Index; CPITN = Community Periodontal Index and Treatment Needs; DAI = Dental Aesthetic Index; DS = Down Syndrome; ASD = Autism Spectrum Disorder; ADHD = Attention deficit hyperactivity disorder; ID = Intellectual Disability; CP = Cerebral Palsy.

**Table 2:** The publication bias and heterogeneity of pooled articles.

Index	Source	Publication Bias	Heterogeneity	Test of Overall Effect	
	No.	Egger’s Test (Two-Tailed)	*I^2^* (%)	*Z*	*p*
DMFT	13	0.08	95.84	8.45	*p* < 0.001
Plaque	4	0.45	98.17	2.39	0.017
CPITN	2	-	-	14.08	*p* < 0.001
OHI-S	5	0.58	93.41	9.91	*p* < 0.001
Gingiva	3	0.04 *	95.77	−2.52	0.012

* *p* < 0.05 represents there are publication bias.

**Table 3:** Subgroup analysis of pooled DMFT index.

Subgroup	No. of Studies	Std Diff in Means	95% CI		Heterogeneity *I*^2^ (%)	*p* Test of Null (2−Tailed)
All studies	13.00	0.44	0.34	0.54	95.84	<0.001
Disability type						
ADHD	1	−0.52	−1.03	−0.01	0	0.047
ASD	3	0.96	0.71	1.20	94.09	<0.001
CP	2	0.06	−0.18	0.30	82.69	0.608
Disability not specified	1	0.96	0.75	1.17	0	<0.001
DS	4	−0.10	−0.30	0.10	97.79	0.309
EP	1	0.15	−0.32	0.62	0	0.543
ID	1	1.41	0.98	1.85	0	<0.001
Country						
China (Hong Kong)	1	0.15	−0.32	0.62	0	0.543
India	6	0.67	0.53	0.82	90.53	<0.001
Israel	2	−1.74	−2.15	1.33	98.38	<0.001
United Arab Emirates	3	0.57	0.39	0.75	96.08	<0.001
Yemen	1	0.38	0.008	0.76	0	0.045
12-Year-Old Children Average DMFT (1994–2014)						
Low <2.5	3	−0.93	−1.23	0.62	97.94	<0.001
Moderate 2.6–3.5	1	0.38	0.01	0.76	0	0.045
High >3.5	9	0.63	0.52	0.75	92.35	<0.001

## Data Availability

The data presented in this study are available on request from the corresponding author.

## Supplementary Materials

The following are available online at https://www.mdpi.com/article/10.3390/healthcare9040410/s1, Figure S1. Pubmed run (Conducted on 28 October 2019 and 15 January 2020); Figure S2. Web of Science Run (Conducted on 28 October 2019 and 15 January 2020); Figure S3. Wiley Run (Conducted on 28 October 2019 and 15 January 2020); Figure S4. Scopus Run (Conducted on 28 October 2019 and 15 January 2020); Figure S5. Cochrane Run (Conducted on 28 October 2019 and 15 January 2020); Figure S6. The world-wide average number of DMFT in 12-year-olds (1994–2014); Figure S7. Publication bias of extracted oral health index data. Table S1. Summary of search results. Table S2. Oral health status among ID children of Asian characteristics included in the studies; Table S3. Quality Assessment of Studies (The Joanna Briggs Institute Critical Appraisal Checklist); Table S4. Risk of bias (ROBINS-E); Table S5 the DMFT index data extracted from included studies; Table S6. Plaque index data extracted from the included studies. Table S7. The oral hygiene index simplified (OHI-S) data were extracted from the included studies; Table S8. The CPITN data were extracted from the included studies; Table S9. Gingival index data extracted from the included studies.
