# Supplementary material for: The Oral Health Inequities between Special Needs Children and Normal Children in Asia: A Systematic Review and Meta-Analysis
Source: Healthcare (Basel). 2021 Apr 2;9(4):410. doi: 10.3390/healthcare9040410 (PMC8065439; doi:10.3390/healthcare9040410)
Supplement: Supplementary file 1 [file healthcare-09-00410-s001.pdf]

healthcare MDPI

Supplementary tables.

Table S1. Summary of Search Results.

Database	Platform	Years Covered	Dates Conducted	Results
Medline	Pubmed	1946–2019	October 28,2019<br>January 15, 2020	1369
Web of Science	WOS	1900–2020	October 28,2019<br>January 15, 2020	310
Wiley Online Library	Wiley	1991–2019	October 28,2019<br>January 15, 2020	633
Elsevier	Scopus	All years–Present	October 28,2019<br>January 15, 2020	10
Cochrane Library	Cochrane	All dates	October 28,2019<br>January 15, 2020	15
Total				2337
Records after duplicates removed				2105

Table S2. Oral Health Status among ID Children of Asia Characteristics Included in Studies.

No	Study	Covariate	Details
1	Lee et al, 2004	dmfs, OHIs	The OHIs Index and total salivary Ig A similar both of DS and general children, but serotype g-s-IgA, and the serotype c-s-IgA greater significantly in DS group.
2	Namal et al, 2007	DMFT	Children with ASD had better dental caries status than healthy children may due to the ASD parents managed their children diet.
3	Davidovich et al, 2010	PI, GI, DMFT	Sialo chemistry analysis showed calcium, sodium, potassium, and chloride levels were significantly higher in the DS population. These conditions may most influential to lower caries rates among DS children.
4	Luppanapornlarp et al, 2010	CPITN, DAI	ASD children had significantly poorer periodontal health than control group. Similar malocclusion was found between both groups. ASD children showed more diastema, spacing, missing teeth, open bites, reverse overjet, and Class II molar relationship than healthy individuals.
5	Jaber, 2011	GI, OHIs, DMFT	ASD children showed higher caries rates, worse oral hygiene and dental treatment needs than healthy control group.
6	Hidas et al, 2013	DMFT, PI	ADHD children showed similar in caries rate, higher plaque index and hyposalivation compared with the control groups.
7	Rai et al, 2012	OHIs, dentition status index.	Similar dental caries status among ASD children and their siblings. In contrast, oral hygiene of ASD children worse than their siblings.
8	Ameer et al, 2012	CPI, PI, OHIs	ID groups showed lower oral hygiene and higher periodontal disease, may due to the lack of understanding, coordination, or muscular limitations.
9	Purohit & Singh, 2012	CPI, DAI, DMFT	Children with disabilities showed poorer oral hygiene with greater calculus deposition, 30% more caries rates and a 60% higher malocclusion compared to control group.
10	Subramaniam et al, 2014	DMFT, OHIs	DS children showed significantly lower the total antioxidant capacity of saliva and higher salivary sialic acid levels. There was an inverse relationship between total antioxidant capacity and dental caries.
11	Sinha et al, 2015	DMFT, OHIs	Cerebral palsy subject had higher caries and poor oral hygiene may due to drooling problem from swallowing effect. CP children have greater Class 2 Angle's malocclusion caused by abnormal alignment of the tongue, lips, and cheeks along with oral habits.

12	Subramaniam et al, 2014	DMFT, OHIs	The higher of dental caries in CP children due to consistency of diet, inadequate nutrition, and poor oral hygiene.
13	Du et al, 2014	PI, GI, dmfs	ASD children had better gingival health and less caries prevalence than control subjects. Both of group showed similar prevalence of malocclusion.
14	Al-Maweri et al, 2015	PI, GI, DMFT	ASD children high prevalence in oral health problem such as poor oral hygiene, gingivitis, fistulae, ulcerative lesions, gingival hyperplasia and cheilitis. The DMFT score was not statistically significant, in contrast the dmft score was significantly higher in ASD children than control group (5.23 vs. 4.06; $p < 0.001$).
15	Radha et al, 2016	CPI, DMFT	ID children had higher value for Decay and Missing teeth, while general children had higher value for Filling teeth.
16	Al Hashmi et al, 2017	DMFT, OHIs	Caries rate was similar between the CP and control subjects. CP subjects had significantly higher of Class II malocclusion, anterior open bite, anterior spacing, and trauma in anterior teeth. In addition, higher frequencies of macroglossia and drooling.
17	Bhandary, 2017	OHIs, DMFT	ASD children similar with their healthy siblings in caries score and OHIs index showed fair with gingival bleeding.
18	Suhaib et al, 2019	Caries, periodontal disease clinical examination	The mother's education had associated with dental caries and periodontal disease in ASD Children. In addition, ASD demonstrated higher caries incidence and dental plaque on anterior teeth. Self-injurious behaviour and bruxism showed in some ASD children.
19	Ghaith, 2019	Angle malocclusion classification, DMFT, OHIs	The DMFT Index, Open bite and Class III Angle's malocclusion were significantly higher in DS than healthy children.
20	Yeung et al, 2019	DMFT, PI, GI, gingival overgrowth index.	Children with epilepsy showed significantly worse gingival health than control children. Epilepsy children who consume more than 1 antiepileptic drug had a higher dental caries prevalence than use mono-antiepileptic drug therapy.

Ig A = Immunoglobulin A; g-s-IgA = g strain S. mutans-specific IgA; c-s-IgA = c strain S. mutans-specific IgA; dmfs = decayed, missing, and filled primary teeth or surfaces; OHIs = Oral Hygiene Index simplified; DMFT = Decay, Missing, Filled Permanent Teeth; PI = Plaque Index; GI = Gingival Index; CPITN = Community Periodontal Index and Treatment Needs; DAI = Dental Aesthetic Index; DS = Down Syndrome; ASD = Autism Spectrum Disorder; ADHD = Attention deficit hyperactivity disorder; ID = Intellectual Disability; CP = Cerebral Palsy.

Table S3. Quality Assessment of studies (The Joanna Briggs Institute Critical Appraisal Checklist).

JBI Checklists	Study																			
	1	2	3	4	5	6	7	8	9	10	11	12	13	14	15	16	17	18	19	20
Were the criteria for inclusion in the sample clearly defined?	1	1	1	1	1	1	1	1	1	1	1	1	1	1	1	1	1	1	1	1
Were the study subjects and the setting described in detail?	1	1	1	1	1	1	1	1	1	1	1	1	1	1	1	1	1	1	1	1
Was the exposure measured in a valid and reliable way?	1	1	1	1	1	1	1	1	1	1	1	1	1	1	1	1	1	1	1	1
Were objective, standard criteria used for measurement of the condition?	1	1	1	1	1	1	1	1	1	1	1	1	1	1	1	1	1	1	1	1

Were confounding factors identified?	1	1	1	1	1	1	1	1	1	1	1	1	1	1	1	1	1	1	1	1
Were strategies to deal with confounding factors stated?	1	1	1	1	1	1	1	1	1	1	1	1	1	1	1	1	1	1	1	1
Were the outcomes measured in a valid and reliable way?	1	1	1	1	1	1	1	1	1	1	1	1	1	1	1	1	1	1	1	1
Was appropriate statistical analysis used?	1	1	1	1	1	1	1	1	1	1	1	1	1	1	1	1	1	1	1	1

Note: Scoring criteria; 1 = study met the criteria, 0 = study did not meet the criteria. 1 = Lee et al, 2004. 2 = Namal et al, 2007. 3 = Davidovich et al, 2010. 4 = Luppanapornlarp et al, 2010. 5 = Jaber, 2011. 6 = Hidas et al, 2012. 7 = Rai et al, 2012. 8 = Ameer et al, 2012. 9 = Purohit & Singh, 2012. 10 = Subramaniam et al, 2014. 11 = Sinha et al, 2004. 12 = Subramaniam et al, 2014. 13 = Du et al, 2010. 14 = Almaweri et al, 2010. 15 = Radha et al, 2016. 16 = Al Hasmi et al, 2017. 17 = Bhandary, 2017. 18 = Suhaib et al, 2017. 19 = Ghaith, 2019. 20 = Yeung et al, 2019.

Table S4. Risk of Bias (ROBINS-E).

**Study**	**Counfounding**	**Selection**	**Exposures**	**Missing Data**	**Measurement of Outcomes**	**Reported Results**	**Overall**
Lee et al, 2004	Moderate	Low	Low	Low	Low	Moderate	Moderate
Namal et al, 2007	Moderate	Low	Low	Low	Low	Moderate	Moderate
Davidovich et al, 2010	Moderate	Low	Low	Low	Low	Moderate	Moderate
Luppanapornlarp et al, 2010	Moderate	Low	Low	Low	Low	Moderate	Moderate
Jaber, 2011	Moderate	Low	Low	Low	Low	Moderate	Moderate
Hidas et al, 2012	Moderate	Low	Low	Low	Low	Moderate	Moderate
Rai et al, 2012	Moderate	Low	Low	Low	Low	Moderate	Moderate
Ameer et al, 2012	Moderate	Low	Low	Low	Low	Moderate	Moderate
Purohit & Singh, 2012	Moderate	Low	Low	Low	Low	Moderate	Moderate
Subramaniam et al, 2014a	Moderate	Low	Low	Low	Low	Moderate	Moderate
Sinha et al, 2004	Moderate	Low	Low	Low	Low	Moderate	Moderate
Subramaniam et al, 2014b	Moderate	Low	Low	Low	Low	Moderate	Moderate
Du et al, 2010	Moderate	Low	Low	Low	Low	Moderate	Moderate
Almaweri et al, 2010	Moderate	Low	Low	Low	Low	Moderate	Moderate
Radha et al, 2016	Moderate	Low	Low	Low	Low	Moderate	Moderate
Al Hasmi et al, 2017	Moderate	Low	Low	Low	Low	Moderate	Moderate
Ghaith, 2019	Moderate	Low	Low	Low	Low	Moderate	Moderate
Yeung et al, 2019	Moderate	Low	Low	Low	Low	Moderate	Moderate

Note: Moderate = the analysis is strong in respect of this domain for a nonrandomized sample but can not be considered comparable with a Well-performed randomized trial; Low = Study is comparable to a well-performed randomized trial in this area.

Table S5. The DMFT Index data extracted from included studies.

No	Study	Country	Subject	Sample Size	Mean	SD	*p* value
1	Davidovich et al, 2010	Israel	DS	70	3.37	0.56	0.012 *
			N	32	5.9	0.8	
2	Jaber, 2011	United Arab Emirates	ASD	61	1.6	0.64	<0.05 *
			N	61	0.6	0.29	
3	Purohit & Singh, 2012	India	Disability	191	2.52	2.61	<0.01 **
			N	203	0.61	1.12	
4	Hidas et al, 2012	India	ADHD	31	2.55	2.293	0.082
			N	30	4.1	3.595	
5	Subramaniam et al, 2013	India	DS	34	1.68	0.69	0.979
			N	34	1.84	1.12	
6	Subramaniam et al, 2014	India	CP	34	1.44	0.58	0.28
			N	34	1.84	1.12	
7	Sinha et al, 2014	India	CP	50	4.11	2.62	0.03 *
			N	50	2.95	2.75	
8	Almaweri et al, 2015	Yemen	ASD	42	2	2.18	NS
			N	84	1.27	1.77	
9	Radha et al 2016	India	ID	50	6.98	2.37	0.005 **
			N	50	3.7	2.27	
10	Bhandary, 2017	India	ASD	30	0.615	0.112	1.000
			N	30	0.556	0.102	
11	Alhasmi et al, 2017	United Arab Emirates	CP	84	2.83	2.86	0.18
			N	125	2.16	2.89	
12	Yeung et al, 2019	Hong Kong	EP	35	0.85	1.61	0.68
			N	35	0.64	1.254	
13	Ghaith et al, 2019	United Arab Emirates	DS	84	3.32	4.62	0.021 *
			N	112	2.16	2.89	

* $p < 0.05$; ** $p < 0.01$; DS = Down Syndrome; N = normal; ASD = Autism Spectrum Disorder; ADHD = Attention deficit hyperactivity disorder; ID = Intellectual Disability; CP = Cerebral Palsy; EP = epilepsy.

Table S6. Plaque index data extracted from included studies.

No	Study	Country	Subject	Sample Size	Mean	SD	*p*-Value
1	Ameer et al, 2012	India	ID	150	1.3	0.47	<0.001 ***
			N	150	0.64	0.51	
2	Du et al, 2014	China (Hong Kong)	ASD	257	0.45	0.24	<0.001 ***
			N	257	0.6	0.27	
3	Almaweri et al, 2015	Yemen	ASD	42	1.5	0.81	0.002 **
			N	84	1.05	0.51	
4	Yeung et al, 2019	China (Hong Kong)	EP	35	9.81	6.828	0.133
			N	35	7.46	4.212	

** $p < 0.01$; *** $p < 0.001$; ID = Intellectual Disability; N = normal; ASD = Autism Spectrum Disorder; EP = epilepsy.

Table S7. The oral hygiene index simplified (OHI-S) data extracted from included studies.

No	Study	Country	Subject	Sample Size	Mean	SD	*p*-Value
1	Subramaniam et al, 2014	India	DS	34	2.72	1.37	0.001 **
			N	34	1.35	0.75	
2	Lee et al, 2004	Korea	DS	19	0.87	0.9	NS
			N	41	0.84	0.37	

3	Subramaniam et al, 2014	India	CP	34	1.58	0.72	0.177
			N	34	1.35	0.75	
4	Alhazmi et al, 2017	United Arab Emirate	CP	84	1.68	1.34	NS
			N	125	1.42	1.14	
5	Ameer et al, 2012	India	ID	150	3.21	1.04	<0.001 ***
			N	150	1.57	1.13	

NS = not significant. ** $p < 0.01$; *** $p < 0.001$; DS = Down Syndrome; N = normal; CP = Cerebral Palsy; ID = Intellectual Disability.

Table S8. The CPITN data extracted from included studies.

No	Study	Country	Subject	Sample Size	Mean	SD	*p*-Value
1	Radha et al 2016	India	ID	50	2.4	0.72	0.067
			N	50	1.08	0.94	
2	Purohit & Singh, 2012	India	Disability not specified	191	3.6	1.5	<0.001 ***
			N	203	2	0.7	

*** $p < 0.001$; ID = Intellectual Disability; N = normal.

Table S9. Gingival Index data extracted from included studies.

No	Study	Country	Subject	Sample Size	Mean	SD	*p*-Value
1	Du et al, 2014	China (Hong Kong)	ASD	257	0.37	0.29	<0.001 ***
			N	257	0.51	0.27	
2	Almaweri et al, 2015	Yemen	ASD	42	1.36	0.84	0.037 *
			N	84	1.02	0.51	
3	Yeung et al, 2019	China (Hong Kong)	EP	35	6.11	6.34	0.003 **
			N	35	1.61	1.66	

* $p < 0.05$; ** $p < 0.01$; *** $p < 0.001$; N = normal; ASD = Autism Spectrum Disorder; EP = epilepsy.

Supplementary Figures.

PubMed on 15/01/2020 2020 for the period 1976 to January 2020

The key words and MeSH were both incorporated into the search using combining terms "AND" and "OR".

1. **Population (462851)**

   "intellectual disability" OR "intellectual impairment" OR "down syndrome" OR "down's syndrome" OR "autism" OR "autistic spectrum disorder" OR "cerebral palsy" OR "epilepsy" OR "ADHD" OR "Attention deficit / hyperactivity disorder" OR "intellectual retardation" OR "mental retardation" OR" mental disability" OR "mental impairment" OR "mental disorder" OR "learning disability" OR "learning difficulty" OR "learning disorder" AND "preschool" OR "children" OR "child" OR "adolescents" OR "teenagers" OR "teens"

2. **Comparison (643005)**

   "healthy" OR "normal" OR "physically fit" OR "unimpaired" OR "good health" OR "good conditions" OR "general health" AND "preschool" OR "children" OR "child" OR "adolescents" OR "teenagers" OR "teens"

3. **Outcome (362611)**

   "oral health status" OR "oral health condition" OR "caries" OR "decay" OR "dmft" OR "oral hygiene" OR "OHIS" OR "periodontal disease" OR "periodontitis" OR "gingivitis" OR "gingival inflammation" OR "CPITN" OR "community periodontal index of treatment needs"

4. **1 AND 2 (96271)**

5. **3 AND 4 (1369)**

Figure S1. Pubmed Run (Conducted on 28 October 2019 and 15 January 2020).

Web of Science on 15/01/2020 for the period 1977 to January 2020

The key words and MeSH were both incorporated into the search using combining terms "AND" and "OR".

1. **Population (607365)**
"intellectual disability" OR "intellectual impairment" OR "down syndrome" OR "down's syndrome" OR "fragile X syndrome" OR autism OR "autistic spectrum disorder" OR "cerebral palsy" OR "Asperger syndrome" OR "intellectual retardation" OR "mental retardation" OR "mental disability" OR "mental impairment" OR "mental retardation" OR "mental disorder" OR "communicative impairment" OR "learning disability" OR "learning difficulty" OR "learning disorder"

2. **Population (646447)**
"preschool" OR "children" OR "child" OR "adolescents" OR "teenagers" OR "teens"

3. **Comparison (1704236)**
"healthy" OR "normal" OR "physically fit" OR "unimpaired" OR "good health" OR "good conditions" OR "general health"

4. **1 AND 2 (124101)**

5. **2 AND 3 (210019)**

6. **4 AND 5 (35495)**

7. **Outcome (59241)**
"oral health status" OR "oral health condition" OR "caries" OR "dmft" OR "oral hygiene" OR "OHIs" OR "periodontal disease" OR "periodontitis" OR "gingivitis" OR "gingival inflammation" OR "CPITN" OR "community periodontal index of treatment needs"

8. **6 AND 7 (310)**

Figure S2. Web of Science Run (Conducted on 28 October 2019 and 15 January 2020).

Wiley on 15/01/2020 for the period 1991 to January 2020

The key words and MeSH were both incorporated into the search using combining terms "AND" and "OR".

1. **Population (2968)**

   "intellectual disability" OR "intellectual impairment" OR "down syndrome" OR "down's syndrome" OR "autism" OR "autistic spectrum disorder" OR "cerebral palsy" OR "epilepsy" OR "ADHD" OR "Attention deficit / hyperactivity disorder" OR "intellectual retardation" OR "mental retardation" OR" mental disability" OR "mental impairment" OR "mental disorder" OR "learning disability" OR "learning difficulty" OR "learning disorder"

2. **Comparison (796175)**

   "healthy" OR "normal" OR "physically fit" OR "unimpaired" OR "good health" OR "good conditions" OR "general health"

3. **Outcome (12067)**

   "oral health status" OR "oral health condition" OR "caries" OR "dmft" OR "oral hygiene" OR "OHIs" OR "periodontal disease" OR "periodontitis" OR "gingivitis" OR "gingival inflammation" OR "CPITN" OR "community periodontal index of treatment needs"

4. **1 AND 2 AND 3 (633)**

Figure S3. Wiley Run (Conducted on 28 October 2019 and 15 January 2020).

Scopus was searched using the Elsevier B.V on 28/03/2019 for the period 1977 to March 2019

The key words and MeSH were both incorporated into the search using combining terms "AND" and "OR".

1. **Population (1251076)**
   "intellectual disability" OR "intellectual impairment" OR "down syndrome" OR "down's syndrome" OR "fragile X syndrome" OR autism OR "autistic spectrum disorder" OR "cerebral palsy" OR "Asperger syndrome" OR "intellectual retardation" OR "mental retardation" OR "mental disability" OR "mental impairment" OR "mental retardation" OR "mental disorder" OR "communicative impairment" OR "learning disability" OR "learning difficulty" OR "learning disorder"

2. **Population (7992272)**
   "preschool" OR "children" OR "child" OR "adolescents" OR "teenagers" OR "teens"

3. **1 AND 2 (773366)**

4. **Comparison (9235075)**
   "healthy" OR "normal" OR "physically fit" OR "unimpaired" OR "good health" OR "good conditions" OR "general health"

5. **2 AND 4 (2112908)**

6. **Outcome (305076)**
   "oral health status" OR "oral health condition" OR "caries" OR "dmft" OR "oral hygiene" OR "OHIs" OR "periodontal disease" OR "periodontitis" OR "gingivitis" OR "gingival inflammation" OR "CPITN" OR "community periodontal index of treatment needs"

7. **3 AND 5 (548)**

8. **6 AND 7 (10)**

Figure S4. Scopus Run (Conducted on 28 October 2019 and 15 January 2020).

Cochrane on 28/03/2019 for the period 1977 to March 2019

The key words and MeSH were both incorporated into the search using combining terms "AND" and "OR".

1. **Population (29009)**
"intellectual disability" OR "intellectual impairment" OR "down syndrome" OR "down's syndrome" OR "fragile X syndrome" OR autism OR "autistic spectrum disorder" OR "cerebral palsy" OR "Asperger syndrome" OR "intellectual retardation" OR "mental retardation" OR "mental disability" OR "mental impairment" OR "mental retardation" OR "mental disorder" OR "communicative impairment" OR "learning disability" OR "learning difficulty" OR "learning disorder"

2. **Population (162608)**
"preschool" OR "children" OR "child" OR "adolescents" OR "teenagers" OR "teens"

3. **1 AND 2 (12954)**

4. **Comparison (231440)**
"healthy" OR "normal" OR "physically fit" OR "unimpaired" OR "good health" OR "good conditions" OR "general health"

5. **2 AND 4 (25290)**

6. **3 AND 5 (1521)**

7. **Outcome (16147)**
"oral health status" OR "oral health condition" OR "caries" OR "dmft" OR "oral hygiene" OR "OHIs" OR "periodontal disease" OR "periodontitis" OR "gingivitis" OR "gingival inflammation" OR "CPITN" OR "community periodontal index of treatment needs"

8. **6 AND 7 (15)**

Figure S5. Cochrane Run (Conducted on 28 October 2019 and 15 January 2020).

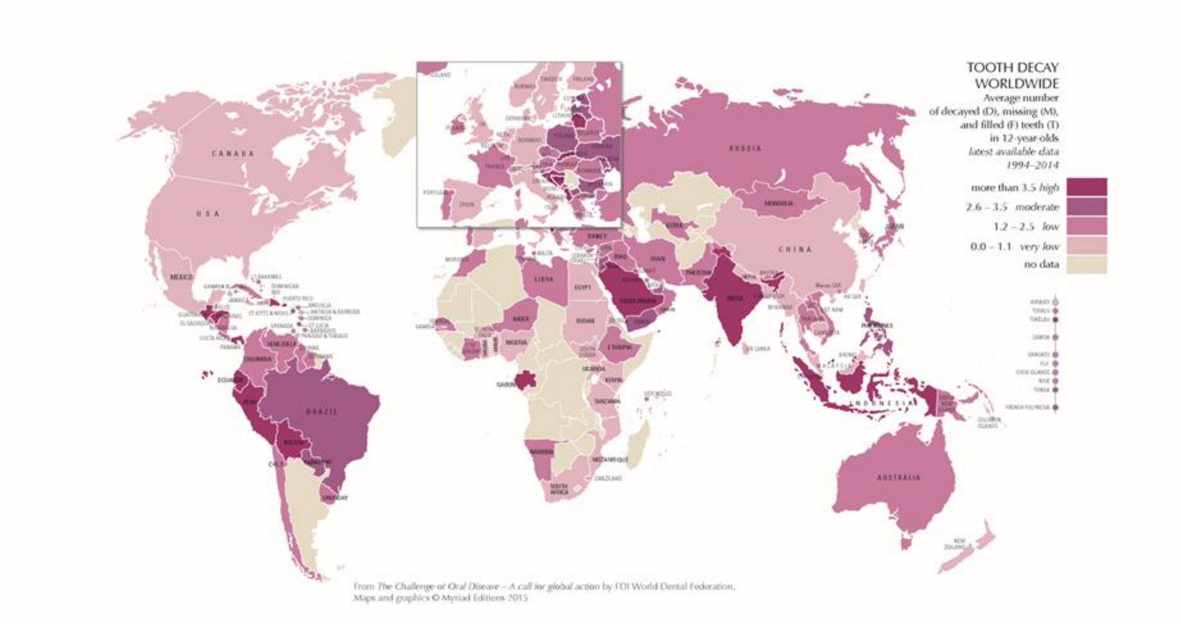

Figure S6. The world-wide average number of DMFT in 12-year-olds (1994–2014). Benzian H, Williams D, eds. The Challenge of Oral Disease – A call for global action. The Oral Health Atlas. 2nd ed. Geneva: FDI World Dental Federation; 2015.

A

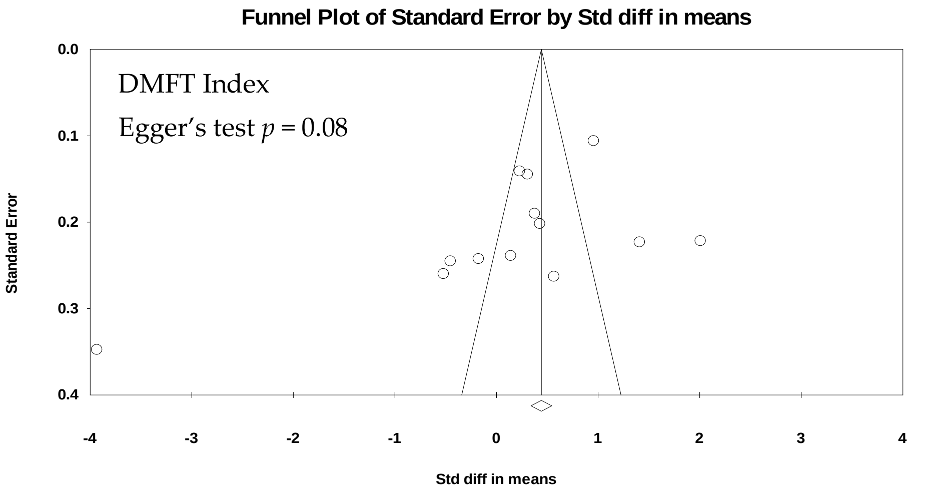

B

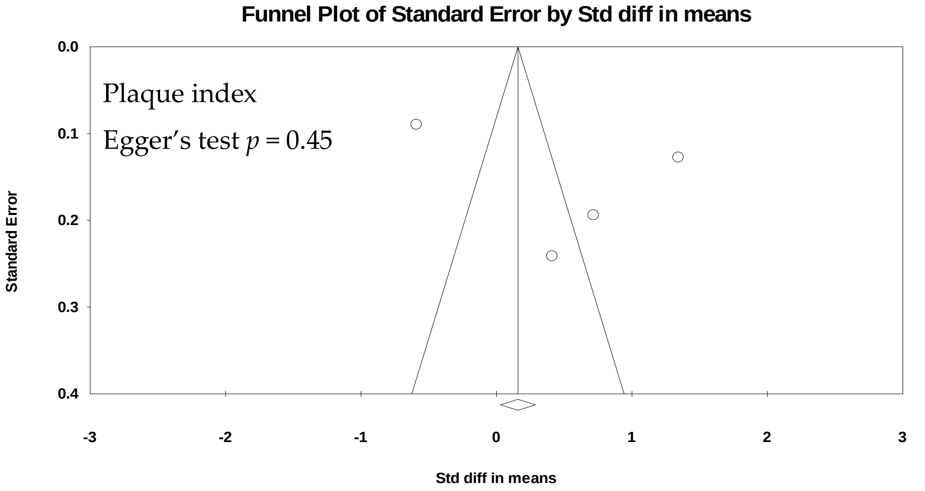

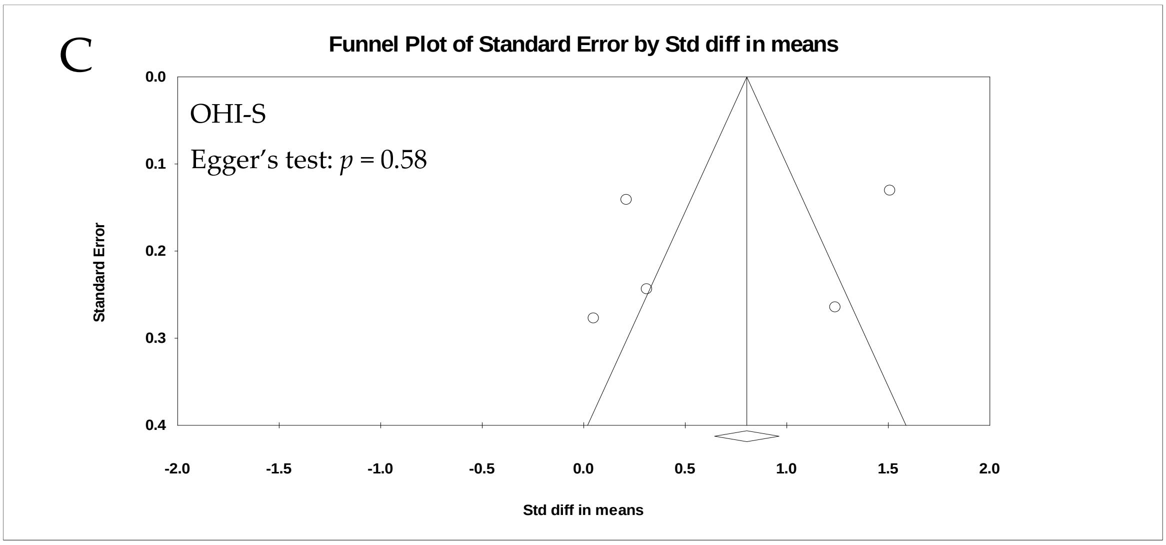

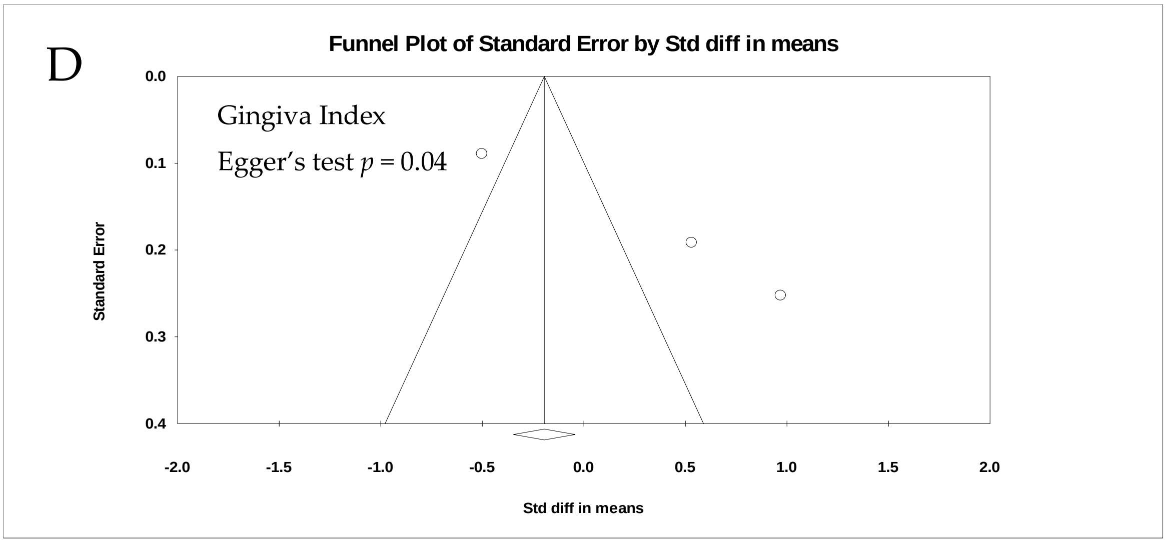

Figure S7. Publication bias of extracted oral health index data. The publication bias was shown by the funnel plot. The X-axis is observed outcomes (standard difference in means). The Y-axis is study precision (standard error). The dot on the plot represents each included study and the upper top indicated the larger or more powerful study. The vertical line in the middle of the funnel indicated the overall effect. (**A**). The funnel plot of the articles included in DMFT index. (**B)**. The funnel plot of the articles included in plaque index. (**C**). The funnel plot of the articles included in OHI-S. (**D**). The funnel plot of the articles included in gingiva index. Egger's test $p < 0.05$ represents publication bias exists.
